# Telehealth vs in-person education for enhancing self-care of ostomy patients (Self-Stoma): Protocol for a noninferiority, randomized, open-label, controlled trial

**DOI:** 10.1371/journal.pone.0303015

**Published:** 2024-06-26

**Authors:** Paolo Iovino, Ercole Vellone, Alessia Campoli, Carmelina Tufano, Maria Rosaria Esposito, Monica Guberti, Tatiana Bolgeo, Cinzia Sandroni, Alessandro Sili, Duilio Fiorenzo Manara, Rosaria Alvaro, Laura Rasero, Giulia Villa

**Affiliations:** 1 Department of Health Sciences, University of Florence, Florence, Italy; 2 Department of Biomedicine and Prevention University of Rome Tor Vergata, Rome, Italy; 3 Department of Nursing and Obstetrics, Wroclaw Medical University, Wroclaw, Poland; 4 Nursing, Technical, Rehabilitation, Assistance and Research Direction, IRCCS Istituti Fisioterapici Ospitalieri, IFO, Rome, Italy; 5 Azienda Ospedaliera San Giuseppe Moscati, Avellino, Italy; 6 Qualità Risk Management-CIO INT IRCCS "Fondazione G. Pascale", Napoli, Italy; 7 Head of Research and EBP Unit, Health Professions Department, Azienda Unità Sanitaria Locale–IRCCS, Reggio Emilia, Italy; 8 Department Attività Integrate Ricerca e Innovazione, Azienda Ospedaliera SS Antonio e Biagio e C. Arrigo, Alessandria, Italy; 9 Albano Laziale, Roma, Italy; 10 Tor Vergata University, Hospital, Rome, Italymailto:; 11 Center for Nursing Research and Innovation, Vita-Salute San Raffaele University, Milan, Italy; Federal University of São João del Rei, BRAZIL

## Abstract

**Introduction:**

Postoperative patients with ostomies experience significant changes in their lives as a result of the device implantation. Self-care is important to improve their health outcomes. Telehealth provides an opportunity to expand access to self-care education.

**Aim:**

This is a multicenter, non-inferiority randomized, open-label, controlled trial to evaluate the non-inferiority of a telehealth intervention to the standard in-person approach in improving self-care behaviors.

**Methods and analysis:**

Three hundred and eighty-four patients aged ≥ 18 years, with a recently placed ostomy, no stomal/peristomal complications, and documented cognitive integrity will be randomly assigned (1:1) to receive either a telehealth intervention (four remote educational sessions) or a standard educational approach (four in-person sessions) delivered in outpatient settings. Every session (remote and in-person) will occur on Days 25, 32, 40, and 60 after discharge. Follow-ups will occur 1, 3, and 6 months after the last intervention session. Primary outcome is self-care maintenance measured using the Ostomy Self-care Index (OSCI). Secondary outcomes include self-care monitoring, self-care management, self-efficacy (OSCI), quality of life (Stoma specific quality of Life), depression (Patient Health Questionnaire-9), adjustment (Ostomy Adjustment Inventory-23), stomal and peristomal complication rates, healthcare services utilization, mobility, and number of working days lost. Analyses will be performed per intention-to-treat and per protocol.

**Ethics and dissemination:**

This study has been approved by the Institutional Review Board of the main center (registration number: 119/22). Following completion of the trial, dissemination meetings will be held to share the results of the study with the participants and the health-care team. Adoption of telehealth technologies for ostomy patients can improve service organization by ensuring better integration and continuity of care. If the remote intervention produces comparable effects to the in-person intervention, it would be wise to make telehealth education an alternative treatment for addressing the educational needs of uncomplicated postoperative ostomy patients.

**Trial registration:**

ClinicalTrials.gov (identifier number: NCT05796544).

## Introduction

Ostomies are the result of a surgical procedure by which an opening is created on the anterior abdomen to eliminate waste (i.e., urine and/or feces) [1]. Ostomies are a common condition globally; in the US, the prevalence is estimated at one million individuals [2], while in Europe approximately 700,000 people live with an ostomy every day [3]. The clinical conditions associated with the presence of an ostomy are cancer, inflammatory bowel diseases, trauma, and familial adenomatous polyposis [4].

Although some types of ostomies are intended to be temporary, the type of disease or other circumstances may require the patient to carry the device for months or even permanently [5]. Changes that start at the preoperative stage and are carried out during the postoperative period can significantly impact various aspects of a patient’s life, including physical, social, and psychological well-being. Physically, patients can experience difficulties in the management of the ostomy and related complications [6, 7]. Socially, individuals may experience sexual function problems, and may also tend to isolate themselves to avoid embarrassing situations in the public [8]. In addition, some patients experience depressive symptoms [9] and exhibit poor quality of life [10]. Traditional ostomy educational interventions have shown substantial effectiveness on various patient outcomes; likewise, studies on telehealth interventions also show to be effective but are poor in number [11–13].

Therefore, the aim of this non-inferiority randomized clinical trial (RCT) is to (i) compare a telehealth intervention to a standard educational approach delivered in outpatient settings under the hypothesis that the telehealth intervention is not inferior to the in-person approach in improving maintenance (primary outcome), monitoring and management self-care behaviors (secondary outcomes); (ii) evaluate the superiority of the telehealth education compared to the in-person sessions, on other secondary outcomes such as quality of life, depression, satisfaction with care, adjustment, rates of stomal and peristomal complications, health care services use, mobility, and number of lost working days, and (iii) report the experience of the patients regarding both the in-person and telehealth education received.

## Background

Ostomy self-care has been shown to improve patient outcomes (e.g., psychosocial adjustment and quality of life) [14, 15]. According to the conceptualization by Villa, Vellone [16], which was in turn, based on the middle-range theory of self-care of chronic illness [17], ostomy self-care is a naturalistic decision-making process that (i) influences the actions linked to the physiological stability of the stoma and peristomal skin, (ii) facilitates the perception of issues and complications, and (iii) orients to the management of them. These behaviors are summarized in the domains of self-care maintenance, monitoring, and management [17]. A fundamental key in the process of self-care is self-efficacy, defined as the belief in one’s abilities to perform self-care practices [16]. Self-efficacy has consistently been shown to be a strong predictor of all self-care activities, and an essential targeted construct of educational interventions in patients with chronic conditions [18].

Although self-care behaviors are important for improving ostomy health outcomes, studies have shown that patients often experience difficulties in performing daily self-care [6, 7]. This has prompted health care providers and researchers to design and implement different educational interventions for these populations. Although the literature shows a certain degree of heterogeneity in intervention studies (e.g., different populations with ostomy and intervention content), pooled results from a systematic review [19] confirm that educational interventions for ostomy patients delivered in the postoperative period transmit substantial improvements in self-care practices (including knowledge), hospitalization time, quality of life, adjustment to the stoma, and other physical mental and social aspects.

As a result of the spread of COVID-19, educational interventions for ostomy patients have undergone substantial revisions in the way they are provided [20]. The priority of avoiding exposure to infection risks has forced healthcare systems to switch to telehealth education. Telehealth can be considered an alternative solution to extend access to education while concomitantly maintaining the level of effectiveness of traditional strategies and keeping patients at home as much as possible [20]. Indeed, telehealth can increase access to care, and is equivalent to in-person services, at least in the specialties of mental health, dermatology, and rehabilitation [21].

## Methods

### Overview

This study will be a two-arm, multicentric, non-inferiority, randomized, open-label, controlled clinical trial with two parallel groups (1:1). Ethical approval has been obtained from the Institutional Review Board of the main center (registration number: 119/22) and registration has been provided at ClinicalTrials.gov (identifier number: NCT05796544) prior to the enrolment of the first participant. The first recruitment occurred on June 14, 2023, and the study is scheduled to end in April 2025. Patients will be randomized into two arms: (1) telehealth education and (2) in-person education.

### Recruitment and eligibility criteria of participants

Three hundred and eighty-four ostomy patients will be recruited from five outpatient settings in northern and central Italy. Study eligibility will be assessed during the visit on day 15^th^ after discharge. Inclusion criteria for eligibility are as follows: (i) having undergone a surgical operation with subsequent ostomy placement; (ii) being able to read and speak the Italian language: (iii) being at least 18 years of age; (iv) absence of cognitive decline, assessed with a score greater than 4 on the Six-Item Screener [22]; (v) absence of any stomal or peristomal complication; (vi) ability to perform a videocall; and (vii) willing to participate in the trial and sign the informed consent form.

### Description of the interventions

**Fig 1:**
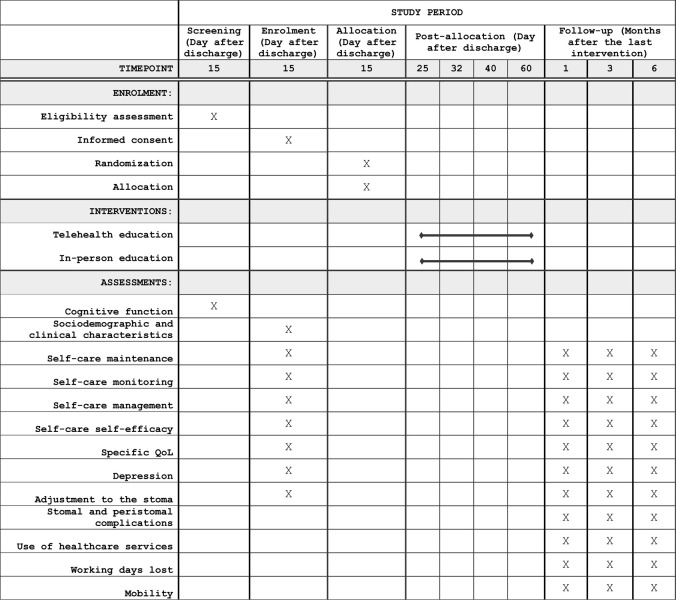
SPIRIT schedule of enrolment, interventions, and assessments of the Self-Stoma study.

The intervention will be performed by enterostomal nurse therapists (ENT) and consists of four remote educational sessions delivered by video calls to patients in their households who underwent a surgical intervention for the placement of urinary or intestinal ostomies. The first session will be delivered the 25^th^ day after discharge from the hospital. The other three sessions will be carried out by the same ENT who will provide the first session and will occur at 32, 40, and 60 days from hospital discharge (Fig 1). This time frame is commonly adopted in Italy for the care of these patients, and the four educational sessions that are part of the intervention are preceded by a stabilization phase, during which all patients are administered two in-person visits during the 7^th^ and 15^th^ days from hospital discharge.

The first session (about 45 min) will cover the following topics: care of the ostomy, elimination, nutrition, complications, and discussion of possible signs and symptoms that necessitate prompt consultation with a healthcare provider. The remaining sessions (each about 45 min) will cover routine stoma care procedures, such as skin care, emptying and replacing the stoma pouch, preventing leakage, and monitoring practices such as interpreting the characteristics of the urine and feces, monitoring body weight, and checking the peristomal skin. Each session will also be tailored to the patients’ educational needs. For example, if they express or show doubts or lack of knowledge on a particular topic, the ENT will spend additional time on it, until the patients demonstrate the doubts have been dissipated.

Patients in the control group will receive the same educational interventions planned for the experimental group, that is, at 25, 32, 40, and 60 days after hospital discharge (Fig 1). However, the visits will be delivered in person at the outpatient setting. In addition, the content of the educational sessions will be identical to that of the experimental group.

The intervention is embedded in the principles of the Teach Back method [23], in which the patient is requested to repeat the instructions that were previously learned during the educational process. For example, the patient is asked to repeat the self-care behaviors necessary for ostomy care or the main signs and symptoms of complications and relative actions in response to these. The teach back method has been shown to have positive effects on a wide range of health outcomes, including adherence to chronic disease self-care [24]. During the visits, the ENT regularly checks the integrity of the stoma and peristomal skin and allows the patient to report possible concerns about any type of problem that arises during the educational process.

### Treatment fidelity assessment

The fidelity of treatment will be documented using a checklist developed according to the principles of the Teach Back method and the theoretical framework.

### Theoretical framework

Bandura’s social cognitive theory [25] was adopted to explain how the teach back method leads to behavioral change. According to this theory, individual performance is inextricably tied to self-efficacy. The level of performance is also affected by the access to health-related information and overall health literacy. Therefore, health literacy can affect health behavior through its influence on self-efficacy. In other words, those with higher health literacy feel more confident about managing their health, because they can use health information appropriately. The middle-range theory of self-care of chronic illness [17], explains how better self-care practices result in better health status, quality of life, health care use, and symptoms.

### Baseline and follow-up assessments

At 15 days from discharge after ostomy placement, the ENT will approach possible participants, ask for their participation, explain the objectives and characteristics of the study, and ask for verbal and written consent. Subsequently, ENTs will screen the patients based on the inclusion criteria. Those who will be considered eligible, will be assessed for sociodemographic (e.g., gender, age), and clinical characteristics (type of ostomy placed, comorbidities). The patients will also be interviewed using a series of psychometrically valid and reliable instruments (Table 1). The same instruments will also be administered 1, 3, and 6 months after the last educational intervention session. Both baseline and follow-up assessments will be performed by a group of specifically trained researchers, who are blinded to the treatment allocation and will be different from the interventionists.

**Table 1:** Variables and instruments used in the trial.

Variable	Instrument	Data source	items	Score range	Validity	Reliability
***General***						
Cognitive function	Six-item screener	Investigator collected	6	0–6	Construct [22]	Cronbach’s alpha: 0.70 [22]
Sociodemographic and clinical characteristics	Questionnaire	Chart review	-	-	-	-
***Primary endpoint***						
Self-care maintenance	OSCI	Self-report	9	0–100	Construct [16]	Cronbach’s alpha: 0.97 [16]
***Secondary endpoints***						
Self-care monitoring	OSCI	Self-report	7	0–100	Construct [16]	Cronbach’s alpha: 0.95 [16]
Self-care management	OSCI	Self-report	5	0–100	Construct [16]	Cronbach’s alpha: 0.93 [16]
Self-care self-efficacy	OSCI	Self-report	10	0–100	Construct [16]	Cronbach’s alpha: 0.96 [16]
Specific QoL	Stoma QoL	Self-report	20	20–80	Construct [26]	Cronbach’s alpha: 0.90 [27]
Depression	PHQ9	Self-report	9	0–27	Construct [28]	Cronbach’s alpha: 0.89 [28]
Adjustment to the stoma	OAI-23	Self-report	23	0–92	Construct validity [29]	Cronbach’s alpha: 0.91 [30]
Rates of stomal and peristomal complications	Questionnaire	Chart review	-	-	-	-
Rates of inappropriate use of healthcare services	Questionnaire	Chart review	-	-	-	-
Number of working days lost	Questionnaire	Self-report	-	-	-	-
Mobility	Questionnaire	Self-report	-	-	-	-

OAI-23, Ostomy Adjustment inventory-23; OSCI, Ostomy Self-care Index; QoL, Quality of Life.

### Randomization and masking

After the screening assessment and baseline data collection (day 15 after hospital discharge), each patient will be sequentially randomized to one of the two intervention conditions, according to a 1:1 allocation ratio. Stratified randomization will be implemented according to gender and the type of ostomy device. The randomization process will occur at the Center of Nursing Research and Innovation of the University of Vita-Salute San Raffaele, Milan, using the REDCAP® software.

Given the intervention’s interactive nature, the ENTs who perform the intervention are not blinded to treatment allocation; however, they will not participate in data collection and analysis. Similarly, researchers and study personnel who will collect and analyze the data will be blinded to patient allocation.

### Outcome measures

#### Primary outcome

The primary outcome will be ostomy self-care maintenance, measured using the Ostomy Self-care Index (OSCI) [16]. The OSCI is a valid and reliable instrument that measures the self-care maintenance, monitoring, and management abilities of ostomy patients. Self-care maintenance is measured with 9 items formulated on a 5-point Likert scale that investigates the frequency of health practices related to maintaining the physiological stability of the stoma and peristomal skin (e.g., cleaning the skin around the stoma and stoma, eating, and drinking according to information received). Total score ranges from 0 to 100, with higher scores indicating better self-care maintenance. The primary outcome of self-care maintenance will be assessed at enrolment (i.e., 15 days after discharge) and 1, 3, and 6 months after the intervention (Fig 1).

#### Secondary outcomes

The secondary outcomes of this study will be evaluated using the following instruments: (i) the Self-care Monitoring, Self-care Management and Self-efficacy scales of the OSCI, to measure the vigilance of the stoma and the stoma skin, the recognition and response to problems detected and the confidence in managing the self-care process; (ii) Stoma Specific Quality of Life (Stoma QoL) [27], to assess the quality of life of sleep, sexual activity, relationship with the family and intimate friends, and social relations outside the family and friends; (iii) Patient Health Questionnaire 9 (PHQ-9) [28], to measure depression, and (iv) Ostomy Adjustment Inventory 23 (OAI-23) [30], to measure the domains of acceptance, negative emotions and social engagement relative to the process of adjustment to the stoma. At each follow-up, patients will be interviewed about the types of stomal and peristomal complications experienced, the relative management (outpatient care or hospital admission), inappropriate use of health care services (number of access to emergency services, number of specialistic surgical visits, and number of admissions), number of working days lost (number of work permits, number of days dedicated to visits, or other), and mobility (distance in km from the patient house to the hospital or vice versa, means of transportation used, presence of a companion). Finally, the “free thoughts”, or an open questionnaire, where the patient can share his or her thoughts about the educational process and its organization. The secondary outcomes will be evaluated at baseline (i.e., 15 days after discharge) and 1, 3, and 6 months after the intervention (Fig 1).

### Validity, reliability and rigor

All the instruments involved in this trial have valid and reliable properties (Table 1). The OSCI has shown adequate Cronbach’s alpha indices for all the three scales (maintenance = 0.965; monitoring = 0.953, management = 0.930, and self-efficacy = 0.962) [16]. Stoma QoL also showed satisfactory internal consistency in patients with colostomy and ileostomy (Cronbach’s alpha = 0.90). The PHQ-9 is also valid and reliable when used in patients with chronic conditions with a Cronbach’s alpha of 0.89 [31]. Finally, the OAI-23 provided a reliability of 0.91 in an Italian sample [30].

### Statistical analysis

#### Sample size

The sample size for the present trial was computed using SampSize® [32]. Assuming 89% power and an alpha error of 2.5%, with a standardized non-inferiority margin of 2.5%, a total sample size of 366 subjects (183 for each arm) is required to demonstrate the noninferiority of the telehealth education compared to the in-person education, relative to the primary outcome (self-care maintenance). Expecting a possible 5% dropout, a final sample size of 384 subjects (192 for each arm) is required.

#### Noninferiority margins

The noninferiority margin established for the primary outcome (self-care maintenance) was 4 points. This corresponds to half of the minimum score considered clinically significant (equivalent to 0.5 standard deviations) on standardized self-care scales [33]. The choice of the non-inferiority margin was derived after careful clinical judgment among the research team and was based on the general recommendation to adopt a smaller value than the minimal clinically significant change and, at the same time, one that would be clinically irrelevant [34].

For exploratory reasons, we also specified the non-inferiority margins for the secondary outcomes. In this case, each analysis will be complemented by post-hoc power estimations to ascertain that the sample size adopted would be sufficient for parameter estimations. For the self-care monitoring, management, and self-efficacy measures, the same non-inferiority margin was adopted, as the difference of 8 points in these scales still represents the minimal clinically significant change [33].

For the secondary outcomes, we adopted a standardized mean difference of 0.50 as the clinically meaningful difference (MCID) [35]; on the PHQ-9 scale, this equals 3.7 points, as derived from a pooled estimation of clinical trials [36]. The resulting non-inferiority margin was set at half this point, or 1.90 points [34].

For Stoma-QoL, we adopted a standardized mean difference of 0.5, as the MCID [35]; assuming a standard deviation of 13 Stoma-QoL points [27], the subsequent MCID was 6.5. The resulting non-inferiority margin was set at half this point, or 3.25 [34]. For OAS-23, we pooled an SD of 13 from observational studies [37–39], and the subsequent MCDI was 6.5. Therefore, the non-inferiority margin was set at 3.25 [34].

The definition of a non-inferiority margin for the prevalence of short-term complications in the intervention and control groups was difficult, as the exact proportion was not known before the start of the trial, and literature regarding rigorous randomized controlled trials from which to extrapolate the data was scarce. However, based on Forsmo, Pfeffer [40], the complication rate obtained one month after an in-person educational intervention was 37,7%. Therefore, we chose to consider 37.7% as the non-inferiority margin.

Unfortunately, readmission rates are unknown after educational interventions performed during RCTs; accordingly, we established the non-inferiority margin by extrapolating data from a cross-sectional study by Rojanasarot [41], who found that at 30 days after discharge, 17% of patients undergoing a post-discharge ostomy support program were readmitted to the hospital due to complications. Therefore, we chose to consider 17% as the non-inferiority margin. Mobility and the number of working days lost will be investigated under the superiority hypothesis, as no inferiority margins have been established for these outcomes.

#### Planned statistical analysis

Sociodemographic and clinical characteristics, as well as the outcome scores will be described using central tendency and variability measures, as well as frequencies and percentages as appropriate. Sources of common method bias of self-reported questionnaires will be investigated with Harman’s single tests and correlation marker techniques. The amount of measurement error will be checked by testing the internal consistency reliability of the scales (e.g., Omega coefficient).

To analyze the effect of the interventions on the randomization groups, longitudinal mixed models will be implemented [42]. The non-inferiority of the experimental treatment to the active control will be confirmed if the inferior bound of the 95% confidence interval is not under the pre-established non-inferiority margin.

Due to the nature of the non-inferiority trials, the analyses will be performed both per “intention to treat” and “per protocol”, according to the CONSORT recommendations [34]. The noninferiority hypothesis will be confirmed only when obtained with both the statistical approaches [43]. Regarding the missing values analyses, the distribution of the subjects lost at follow-up will be compared between the two randomization groups, to assess possible differences in sociodemographic and clinical characteristics or outcomes. The results will be interpreted considering the possible differences between the groups. In all the analyses, values less than 0.05 will be considered statistically significant. To ensure rigor, EQUATOR guidelines including SPIRIT [44] and CONSORT [45] will guide the reporting process of all analyses. In order to minimize bias, the analysis plan was finalized prior to first participant recruitment.

## Discussion

This non-inferiority RCT aims to understand whether a telehealth intervention is at least as effective as a standard in-person educational approach in improving self-care. The study will also allow us to investigate whether the telehealth intervention is not inferior to standard education in improving the secondary outcomes of quality of life, depression, satisfaction with care, adjustment, rates of stomal and peristomal complications, healthcare services utilization, mobility, and number of lost working days. Another aim of the trial will be also to explore the experience of patients with respect to the education received.

During the COVID-19 pandemic, profound changes have been initiated to rethink the delivery of health education [46]. This has also involved the care of ostomy patients, since many of them are older people affected by chronic conditions, and associated risk factors (e.g., obesity, and hypertension), and thus at a higher risk of developing serious diseases from COVID-19 [20].

The studies conducted so far have been shown to be effective in patients undergoing an ostomy. For example, Augestad, Sneve [11] implemented a 12-month teleconsultation after initial surgery, and found that the readmission rates and burden of travel were significantly reduced, although quality of life did not improve. Iqbal, Raza [13] administered daily telephone calls to ileostomy patients for 3 weeks after discharge, and although using a historical control cohort as a comparison group, they found a reduction in dehydration-related readmission rates and a decrease in hospital costs.

So far, this is the first study to explore the comparative effectiveness of remote and in-person education in ostomy patients. If our study hypothesis proves to be true (i.e., telehealth education is as effective as in-person education), the use of telehealth could integrate or even replace the current practices of in-person delivery. This would also be timely, because it would adapt to the past pandemic, where traditional in-person instructions can carry the risk for individuals to spread viruses to others. We strongly believe that telehealth approaches will continue to expand even after the COVID-19 pandemic, in order to maintain the undeniable advantages that older adults will gain, such as those with problems of transportation, social isolation, physical impediments, and those who live at great distances from the health service. Furthermore, the adoption of telehealth technologies will be able to improve service organization by shifting activities from the hospital to the primary care network or home care, ensuring better integration and continuity of care. As such, it is essential to expand the knowledge of existing research to confirm that remote educational approaches are as effective as traditional ones.

## Conclusion

The prevalence of ostomy patients is expected to increase in the future. Telehealth education has the potential to be as effective as (or superior to) traditional in-person education, leading to positive impacts on health outcomes. If this is confirmed, this study will help inform healthcare providers and researchers tailor future educational interventions in favor of relationship quality, safety, and other satisfaction-related outcomes.

## Supporting information

S1 Checklist SPIRIT 2013 checklist: Recommended items to address in a clinical trial protocol and related documents*. (DOC)

S1 File (XLSX)

S1 Protocol (DOCX)
