# Supplementary material for: Telehealth vs in-person education for enhancing self-care of ostomy patients (Self-Stoma): Protocol for a noninferiority, randomized, open-label, controlled trial
Source: PLoS One. 2024 Jun 26;19(6):e0303015. doi: 10.1371/journal.pone.0303015 (PMC11206953; doi:10.1371/journal.pone.0303015)
Supplement: S1 Protocol — (DOCX) [file pone.0303015.s003.docx]

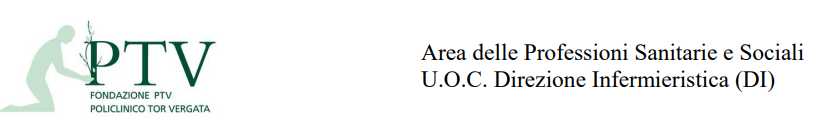


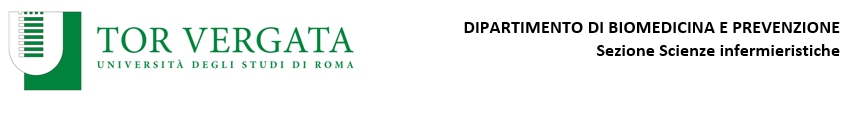


**Remote self-care education in ostomy patients: study protocol for a multicentre randomised, controlled, non-inferiority, parallel trial (SELF-STOMA study).**

**Principal Investigator**: Dr. Alessandro Sili

**Scientific director**: Prof. Ercole Vellone

**Research group**: Prof.ssa Rosaria Alvaro, Prof. Ercole Vellone, Dott.ssa Giulia Villa, Dott.ssa Vittoria Giordano, Dott. Francesco Corvese, Dott. Paolo Iovino, Dott.ssa Alessia Campoli, Dott.ssa Cinzia Sandroni, Dott.ssa Caterina Schirò, Dott. Rocco Paradiso, Dott. Carlo Orru, Dott.ssa Maria Rosaria Esposito, Dr.ssa Tatiana Bolgeo, Dr.ssa Monica Guberti.

**Research protocol**

**INTRODUCTION**

An ostomy is the result of a surgical procedure whereby an external branch is created in the front part of the abdomen to allow faeces or urine to leak out^1^. Ostomies are a common condition worldwide; in America only, the estimated prevalence is one million individuals,^2^ while in Europe, approximately 700,000 individuals live with an ostomy every day.^3^ Conditions associated with the presence of an ostomy are variable; the most common include cancer, chronic inflammatory bowel disease, trauma and familial polyposis adenomatosis.^4^ Due to their widespread prevalence worldwide, cancer of the rectum and of the bladder are the conditions most frequently associated with the presence of an ostomy.^5^

Although some types of ostomy are meant to be temporary, some types of disease or other circumstances allow the patient to live with this device for months, years, or even a lifetime.^6^ The psychological changes that occur during the preoperative phase and continue into the postoperative period can negatively impact various aspects of the patient's life, such as physical and social well-being. On a physical level, patients may experience difficulties with sexual function and often tend to isolate themselves to avoid embarrassing situations in public.^7^ In addition, some patients also show symptoms of depression ^8^ and a poor overall quality of life.^9^

Self-care in ostomy patients has been shown to improve health outcomes such as psychosocial adjustment and quality of life).^10,11^ According to the conceptualisation of self-care provided by Villa, Vellone, Sciara, Stievano, Proietti, Manara, Marzo, Pantaleo,^12^ itself based on the middle-range theory of self-care in chronic diseases,^13^ Self-care in ostomy patients is a naturalistic process of decision-making that (i) influences actions related to the psychological stability of the ostomy and peristomal skin (ii), facilitates the perception of related problems and complications (iii), and directs the management of these. These behaviours are summarised in the domains of self-care maintenance, self-care monitoring, and self-care management, respectively.^13^ A key aspect in the self-care process is self-efficacy, defined as confidence in one's ability to perform self-care behaviours.^12^ The construct of self-efficacy has proven to be a strong predictor of all self-care behaviours and consequently, an important target for psychoeducational interventions in patients with chronic illnesses.^14^

Although self-care behaviours are essential to improve health outcomes in ostomy patients, studies have shown that these individuals have difficulty performing these daily practices ^15,16^. This has prompted healthcare professionals to design and implement educational interventions for this population. Although the literature shows a certain level of heterogeneity of intervention-type studies (e.g. due to differences in characteristics between enroled samples or different content of the interventions), the results of a recent systematic review ^17^ confirm that interventions in the postoperative period for ostomy patients are capable of improving self-care practices (including knowledge), reducing hospitalisation time, quality of life, adaptation to the ostomy, and other aspects of physical, mental and social health.

Recently, due to the spread of Coronavirus 19 (COVID-19) and the subsequent pandemic, educational interventions aimed at ostomy patients have been rethought with respect to their mode of administration.^18^ Indeed, the priority of avoiding exposure to COVID-19 infection has prompted healthcare systems to move to telemedicine and remote technologies. Remote education can be conceived as an alternative solution to expand access to education, while maintaining the level of effectiveness of traditional strategies and urging the patient to stay at home as long as possible.^18^ In this sense, the literature shows how telemedicine can increase access to care, and how it is equivalent to services provided in person, at least in the specialities of mental health, dermatology and rehabilitation.^19^

Traditional educational interventions for patients with ostomy have shown substantial effectiveness in health outcomes; similarly, telemedicine-based education has also been shown to be effective, but studies in this regard are few and far between.^20-22^ The coronavirus pandemic has stimulated the use of telemedicine for patient education, but at present we do not have evidence that remotely administered educational interventions in patients with intestinal or urinary ostomy are equally effective as those delivered in the presence. If remote education was as effective as in-patient education, it could be used routinely, reserving in-patient visits for complications that cannot be managed remotely. Furthermore, if remote ostomy patient education was not less effective than inpatient education, there would be many benefits for patients, such as no need to travel to specialist centres for follow-up visits, less stress due to transport, and less absence from work for follow-up visits. Furthermore, patients living far from specialist centres could receive the same educational interventions in ostomy care as patients living closer to them.

Therefore, the objective of this randomised controlled trial (RCT) is to:

1. Evaluate the non-inferiority of an educational intervention carried out remotely (via video call) on self-care maintenance (primary endpoint), self-care monitoring and self-care management (secondary endpoints) in people with bowel and urinary ostomy compared to the same intervention carried out in the presence;
2. to evaluate the non-inferiority of an educational intervention carried out remotely (via video call) to persons with intestinal and urinary ostomy on other secondary outcomes such as quality of life, depression, satisfaction with care, adaptation, prevalence of ostomal and peristomal complications, use of health care services, mobility, and number of working days lost, compared to the same intervention carried out in the presence;
3. describe the experience of patients who received the educational intervention performed remotely (via video call) and in presence.

**METHODS**

*Study design*

Two-arm interventional study (Figure 1). The study protocol will be approved at each participating centre and will be registered on ClinicalTrials.gov. The coordinating centre will be the “UOC Direzione Infermieristica” of the Policlinico Tor Vergata.

*Intervention*

The intervention will be educational, carried out by stomatherapists, will focus on promoting self-care in the person with an ostomy and will be carried out remotely, by video call, in patients undergoing placement of an intestinal or urinary ostomy from day 25 after hospital discharge. The intervention will be inspired by the Teach Back principles,^23^ a technique whereby the patient is first taught a specific care practice (e.g. how to perform ostomy and peristomal skin care) and then asked to repeat the learnt instructions. The Teach Back method has been proven to be effective in various health outcomes, including the self-care of chronic diseases.^24^ The procedure will be performed by the same stomatherapist who followed the patient during the perioperative period and will be performed in 4 sessions, each lasting approximately 30 minutes, at intervals of 25, 32, 40 and 60 days after hospital discharge following ostomy placement. These timings are those commonly followed by ostomatherapists for the follow-up of patients after ostomy placement and are subsequent to two follow-up visits that are made in the presence 7 and 15 days after hospital discharge. Thus, the experimental intervention would not begin immediately after the patient's discharge but after his "stabilisation" with in-presence follow-ups at 7 and 15 days after discharge.

*Adherence to treatment*

Adherence to treatment will be documented using a checklist developed according to the principles of Teach Back and the theoretical framework. Furthermore, the researchers will ensure that all intervention sessions (at 25, 32, 40 and 60) are carried out as per the research protocol.

*Control group*

The patients in the control group will receive the same educational interventions as in the experimental group, at 25, 32, 40 and 60 days after discharge and using the Teach Back method, but in presence. The educational content will also be the same as in the experimental group.

*Theoretical framework*

Bandura's socio-cognitive theory ^25^ was adopted to explain the mechanism through which Teach Back leads to modification of self-care behaviour. This theory postulates that an individual's performance is intimately linked to his or her self-efficacy. Performance is also intimately influenced by access to health information and health literacy that are improved with the Teach Back intervention. Thus, health literacy can influence health behaviour through its influence on self-efficacy. In other words, individuals with a higher health literacy will feel more confident in managing their health, as they will be able to use information more appropriately. The effect of self-care on health outcomes is explained by the mid-range theory of self-care in chronic diseases,^13^ in which improved health behaviour can in turn lead to improved outcomes (e.g. quality of life, utilisation of health services, symptoms).

*Recruitment and assessment of participant eligibility*

Patients will be recruited from six outpatient nursing facilities in central and northern Italy (see Annex), where ostomatherapy activities are present, 15 days after hospital discharge following the packing of an intestinal or urinary ostomy.

The inclusion criteria will be the following: (i) be at least 18 years old; (ii) ambosexual; (iii) have undergone surgery for the packaging of an intestinal or urinary ostomy; (iv) be able to speak and write in Italian; (v) absence of cognitive decline, assessed by a score greater than 4 on the Six Item Screener questionnaire;^26^ (vi) absence of stomal or peristomal complications; (vii) be able to make video calls from a smartphone or PC; and (viii) willingness to participate in the study and sign written informed consent.

Exclusion criteria will be: (i) minor age; (ii) incomprehension of written and spoken Italian; (iii) cognitive impairment; (iv) stomal complications that make an in-person visit necessary; (v) inability to make video calls (from mobile phone or computer); (vi) refusal to take part in the study.

*Assessment al baseline e ai follow-up*

At 15 days after hospital discharge after intestinal or urinary ostomy placement (baseline), the stomatherapists, in the six centres participating in the study, will identify possible candidates, propose the study to them, inform them, and ask them to sign the informed consent. They will then screen the patients, according to inclusion / exclusion criteria. Patients who pass the screening will be evaluated for sociodemographic (e.g. age, sex) and clinical characteristics (type of ostomy packing and comorbidities, using the Charlson Comorbidity Index ^27^). Patients who do not pass the screening will not be enroled in the study and will be cared for according to common clinical practice.

Patients will also be interviewed using validated instruments (questionnaires and psychometric scales) investigating self-care behaviours, quality of life, depression, adaptation, stomal and peristomal complications, number of inappropriate accesses to health services, lost working days, and mobility (Table 2 and appendices). These same variables will subsequently be reevaluated in both the experimental and control arms at 1, 3 and 6 months after the end of the intervention.

The data collection process will be carried out by specially trained research nurse assistants, who will not know the randomisation arm to which patients have been assigned.

*Randomization and blinding*

After enrolment (at 15 days after discharge) and data collection at baseline, patients will be assigned to the two planned study arms by randomisation stratified by type and sex of ostomy. Randomisation will be implemented in blocks of 6 patients to ensure the same number of individuals per arm. Randomisation blocks will be created until the predetermined sample size is reached. Then, again causally, the blocks will be randomly allocated to enrolment centres.

The stomatherapist who will perform the educational intervention will know the allocation of patients to the two groups, but will not participate in data collection and analysis. Therefore, in this study, the researchers and research assistants who will collect endpoint data will be blinded, and this will also apply to those who will perform data collection and analysis.

*Measurement of outcomes*

Primary outcome. The primary outcome of this trial is self-care maintenance, assessed with the Ostomy Self-Care Index (OSCI) 12 Self-Care Maintenance Scale at 1 month after completion of the educational intervention performed by the ostomaterapists. The OSCI has 32 items grouped into four sections with 5-point Likert scale responses that assess the dimensions of self-care maintenance, self-care monitoring, self-care management and self-care efficacy. The section of self-care maintenance consists of nine items designed to assess daily behaviours for maintaining the physiological stability of the ostomy and peristomal skin (e.g., cleaning the ostomy and properly changing the collection device, following a diet according to the information received). The self-care maintenance section scores range from 0 to 100 where higher scores indicate better self-care behaviours. Self-care maintenance will be assessed at baseline (i.e., 15 days after discharge) in both the experimental and control groups. It will then be assessed at each follow-up (3 and 6 months after the end of the intervention) in both the experimental and control groups (Table 2).

Secondary Outcomes. Secondary outcomes will be assessed with the following instruments: (i) OSCI12's Self-Care Monitoring Scale, Self-Care Management Scale, and Self-Care Self-Efficacy Scale, to measure ostomy and peristomal skin monitoring behaviours, recognition of and response to identified ostomy problems, and self-efficacy in managing the self-care process; each of these scales has a score from 0 to 100 where higher scores indicate better self-care monitoring behaviours, and management, and better self-efficacy, respectively. (ii) Stoma Specific Quality of Life (Stoma QoL),^28^ to assess quality of life in the domains of sleep, sexual activity, relationship with family and close friends, and social relationships outside of family and friends; items in this questionnaire use a 4-point Likert scale varying from 1 (always) to 4 (not at all), and the total score ranges from 20 to 80 where higher scores indicate higher levels of quality of life. (iii) Patient Health Questionnaire 9 (PHQ-9),^29^ to measure depression; the questionnaire consists of 9 items with a 4-point Likert scale, ranging from 0 (not at all) to 3 (almost every day). The score ranges from 0 to 27 points, and the recommended cut-off point for identifying depressive disorders is a score greater than 9 points. (iv) Ostomy Adjustment Inventory-23 (OAI-23),^30^ to measure the domains of acceptance, negative emotions and social involvement relative to ostomy adjustment. The instrument consists of 23 items divided into three dimensions (acceptance, negative feelings and social engagement). Each item uses a 5-point Likert scale from 0 (strongly disagree) to 4 (strongly 'agree). A high score indicates a better level of adjustment. Finally, an unstructured questionnaire called "free thoughts" will be used, where patients in both the experimental and control groups can report their experience with the education they received. Furthermore, at each follow-up data collection (1, 3, and 6 months after the educational intervention) patients will be surveyed on the frequency and type of stomal and peristomal complications, their management (access to emergency services, number of specialist visits, and number of hospitalisations), the number of lost work days (number of work leave, number of days devoted to visits, or other), and mobility (distance in kilometres from home to hospital or vice versa, means of transportation used and presence of a companion). All secondary outcomes will be assessed at baseline and during all follow-ups (Table 2).

*Statistical analysis*

Sample size. The sample size of the present study was calculated with Sampsize.31 Assuming a power of 80% and an alpha error of 2.5%, for a standardised non-inferiority margin equivalent to 0.25, a sample size of 366 subjects (183 for each arm) will be needed to demonstrate non-inferiority of the intervention by remote versus standardised means, relative to the primary outcome (self-care maintenance). Estimating a possible dropout of 10% at 1 month after the end of the educational intervention, it will be necessary to enrol 384 subjects (192 for each arm). Considering that there will be six centres participating in the study, each centre will enrol 64 patients (32 per arm).

Non-inferiority margins. The non-inferiority margin established for the primary outcome (self-care maintenance) will be four points. This margin corresponds to half of the minimum score considered clinically significant (equivalent to 0.5 standard deviations) on the standardized scales of self-care^32^. The choice of this margin of inferiority arose after careful clinical judgment of the research team and based on the general recommendation to adopt a value that is smaller than the minimum clinically significant score and is, at the same time, clinically irrelevant ^33^.

For secondary outcomes self-care monitoring, self-care management and self-efficacy, the same margin of non-inferiority is established, a difference of 8 points being valid in these scales as the minimum clinically significant score ^32^.

Planned statistical analyses. Sociodemographic and clinical characteristics, as well as outcome scores, will be described by measures of central tendency (mean or median) and variability (standard deviation or interquartile range), while frequencies and percentages will be used for categorical variables. To investigate differences in sociodemographic and clinical characteristics between randomisation groups, the t test for independent samples and the chi-square test will be used. P values less than 0.05 will be considered statistically significant. To investigate differences in the primary outcome and secondary outcomes of non-inferiority, the student's t test for independent samples will be employed at each follow-up and baseline. A longitudinal linear mixed model will be implemented to analyze the effect of interventions on randomization groups and their interaction with time. This approach, widely used as a substitute for ANOVA (in order to avoid loss of subjects due to missing data), has the advantage of controlling for the phenomenon of "clustering" induced by repeated measures over time for each subject.^34^

Non-inferiority of the experimental intervention remotely compared with the in-presence intervention will be confirmed if the lower extreme of the 95% confidence interval is not below the predetermined non-inferiority margin. Any superiority of the experimental intervention over the active control will be confirmed if the lower end of the 95% confidence interval is simultaneously above the non-inferiority margin and above zero. Given the nature of non-inferiority trials, analyses will be conducted in both intention-to-treat and per-protocol modes, according to CONSORT recommendations.^33^ The hypothesis of non-inferiority will be confirmed only if obtained through both modes of analysis.^35^ For missing data analyses, the distributions of patients lost to follow-up between the two randomisation groups will be compared (using chi-square tests) in order to assess any differences in sociodemographic and/or clinical characteristics, or outcomes. The results obtained will be interpreted in light of any differences found between the groups.

**CONCLUSIONS**

The prevalence of ostomy patients is likely to increase in the future due to an aging population and an increase in the prevalence of chronic diseases. Self-care education delivered remotely has the potential to be on par (or superior) in effectiveness with traditional education delivered in person, possibly resulting in improved health outcomes. If this hypothesis were to be confirmed, it would be appropriate to make remote education the treatment of choice to meet the educational needs of ostomy patients in the postoperative period, and as a response to the changes originating from the COVID-19 pandemic.

**BIBLIOGRAPHY**

1. Tsujinaka S, Tan KY, Miyakura Y, et al. Current Management of Intestinal Stomas and Their Complications. *J Anus Rectum Colon.* 2020;4(1):25-33.

2. Maydick-Youngberg D. A Descriptive Study to Explore the Effect of Peristomal Skin Complications on Quality of Life of Adults With a Permanent Ostomy. *Ostomy Wound Manage.* 2017;63(5):10-23.

3. Claessens I, Probert R, Tielemans C, et al. The Ostomy Life Study: the everyday challenges faced by people living with a stoma in a snapshot. *Gastrointestinal Nursing.* 2015;13(5):18-25.

4. Burch J. Exploring the conditions leading to stoma-forming surgery. *Br J Nurs.* 2005;14(2):94-98.

5. Bray F, Ferlay J, Soerjomataram I, Siegel RL, Torre LA, Jemal A. Global cancer statistics 2018: GLOBOCAN estimates of incidence and mortality worldwide for 36 cancers in 185 countries. *CA Cancer J Clin.* 2018;68(6):394-424.

6. Ambe PC, Kurz NR, Nitschke C, Odeh SF, Moslein G, Zirngibl H. Intestinal Ostomy. *Dtsch Arztebl Int.* 2018;115(11):182-187.

7. Sun V, Grant M, McMullen CK, et al. Surviving colorectal cancer: long-term, persistent ostomy-specific concerns and adaptations. *J Wound Ostomy Continence Nurs.* 2013;40(1):61-72.

8. Hwang JH, Yu CS. Depression and resilience in ulcerative colitis and Crohn's disease patients with ostomy. *Int Wound J.* 2019;16 Suppl 1:62-70.

9. Geng Z, Howell D, Xu H, Yuan C. Quality of Life in Chinese Persons Living With an Ostomy: A Multisite Cross-sectional Study. *J Wound Ostomy Continence Nurs.* 2017;44(3):249-256.

10. Xian H, Zhang Y, Yang Y, Zhang X, Wang X. A Descriptive, Cross-sectional Study Among Chinese Patients to Identify Factors that Affect Psychosocial Adjustment to an Enterostomy. *Ostomy Wound Manage.* 2018;64(7):8-17.

11. Zhang Y, Xian H, Yang Y, Zhang X, Wang X. Relationship between psychosocial adaptation and health-related quality of life of patients with stoma: A descriptive, cross-sectional study. *J Clin Nurs.* 2019;28(15-16):2880-2888.

12. Villa G, Vellone E, Sciara S, et al. Two new tools for self-care in ostomy patients and their informal caregivers: Psychosocial, clinical, and operative aspects. *International Journal of Urological Nursing.* 2019;13(1):23-30.

13. Riegel B, Jaarsma T, Stromberg A. A middle-range theory of self-care of chronic illness. *ANS Adv Nurs Sci.* 2012;35(3):194-204.

14. Eller LS, Lev EL, Yuan C, Watkins AV. Describing Self-Care Self-Efficacy: Definition, Measurement, Outcomes, and Implications. *International Journal of Nursing Knowledge.* 2018;29(1):38-48.

15. Altschuler A, Liljestrand P, Grant M, Hornbrook MC, Krouse RS, McMullen CK. Caregiving and mutuality among long-term colorectal cancer survivors with ostomies: qualitative study. *Support Care Cancer.* 2018;26(2):529-537.

16. Bulkley JE, McMullen CK, Grant M, Wendel C, Hornbrook MC, Krouse RS. Ongoing ostomy self-care challenges of long-term rectal cancer survivors. *Support Care Cancer.* 2018;26(11):3933-3939.

17. Costa A, Maria M, Campos C, Santos JD. Effect of educational intervention in postoperative people with intestinal elimination stomies: systematic review. 2020.

18. Maculotti D, Spena PR, Villa G. Position Statement on Care of Ostomy Patients during Covid-19 Pandemic. *Gastroenterol Nurs.* 2020;43(4):324-326.

19. Shigekawa E, Fix M, Corbett G, Roby DH, Coffman J. The Current State Of Telehealth Evidence: A Rapid Review. *Health Aff (Millwood).* 2018;37(12):1975-1982.

20. Augestad KM, Sneve AM, Lindsetmo RO. Telemedicine in postoperative follow-up of STOMa PAtients: a randomized clinical trial (the STOMPA trial). *BJS (British Journal of Surgery).* 2020;107(5):509-518.

21. Iqbal A, Raza A, Huang E, Goldstein L, Hughes SJ, Tan SA. Cost Effectiveness of a Novel Attempt to Reduce Readmission after Ileostomy Creation. *JSLS.* 2017;21(1).

22. Weinstein RS, Holcomb MJ, Mo J, et al. An Ostomy Self-management Telehealth Intervention for Cancer Survivors: Technology-Related Findings From a Randomized Controlled Trial. *J Med Internet Res.* 2021;23(9):e26545.

23. Yen PH, Leasure AR. Use and Effectiveness of the Teach-Back Method in Patient Education and Health Outcomes. *Fed Pract.* 2019;36(6):284-289.

24. Ha Dinh TT, Bonner A, Clark R, Ramsbotham J, Hines S. The effectiveness of the teach-back method on adherence and self-management in health education for people with chronic disease: a systematic review. *JBI Database System Rev Implement Rep.* 2016;14(1):210-247.

25. Bandura A. Social foundations of thought and action: A social cognitive theory. In. *Social foundations of thought and action: A social cognitive theory.*: Prentice-Hall, Inc; 1986:xiii, 617-xiii, 617.

26. Callahan CM, Unverzagt FW, Hui SL, Perkins AJ, Hendrie HC. Six-item screener to identify cognitive impairment among potential subjects for clinical research. *Med Care.* 2002;40(9):771-781.

27. Quan H, Li B, Couris CM, et al. Updating and validating the Charlson comorbidity index and score for risk adjustment in hospital discharge abstracts using data from 6 countries. *Am J Epidemiol.* 2011;173(6):676-682.

28. Canova C, Giorato E, Roveron G, Turrini P, Zanotti R. Validation of a stoma-specific quality of life questionnaire in a sample of patients with colostomy or ileostomy. *Colorectal Dis.* 2013;15(11):e692-698.

29. Kroenke K, Spitzer RL, Williams JB. The PHQ-9: validity of a brief depression severity measure. *J Gen Intern Med.* 2001;16(9):606-613.

30. Dellafiore F, Conte G, Baroni I, et al. Ostomy Adjustment Inventory-23 (OAI-23): Development and Testing of the Italian Version. *J Wound Ostomy Continence Nurs.* 2019;46(1):38-43.

31. Flight L, Julious SA. Practical guide to sample size calculations: non-inferiority and equivalence trials. *Pharm Stat.* 2016;15(1):80-89.

32. Riegel B, Lee CS, Dickson VV, Carlson B. An update on the self-care of heart failure index. *J Cardiovasc Nurs.* 2009;24(6):485-497.

33. Piaggio G, Elbourne DR, Pocock SJ, Evans SJ, Altman DG, Group C. Reporting of noninferiority and equivalence randomized trials: extension of the CONSORT 2010 statement. *JAMA.* 2012;308(24):2594-2604.

34. Bell ML, Rabe BA. The mixed model for repeated measures for cluster randomized trials: a simulation study investigating bias and type I error with missing continuous data. *Trials.* 2020;21(1):148.

35. Tripepi G, Chesnaye NC, Dekker FW, Zoccali C, Jager KJ. Intention to treat and per protocol analysis in clinical trials. *Nephrology.* 2020;25(7):513-517.

**Figure 1.** Study flow chart

Patient eligibility assessment

(15 days after hospital discharge)

Baseline assessment of patients

Follow up Assessment of primary and secondary endpoints at 1, 3 and 6 months after surgery

Follow up Assessment of primary and secondary endpoints at 1, 3 and 6 months after surgery

Randomisation

Standard of care:

4 in presence educational interventions, carried out at 25, 32, 40 e 60 days after discharge

Arm 2: Control

arm

Intervention:

4 remotely educational interventions, via videocall, carried out at 25, 32, 40 e 60 days after discharge

Arm 1: Experimental

arm

**Table 2**. Variables and tools

| Variables | Tool | Time of Data Collection  (after educational intervention) | | | |
| --- | --- | --- | --- | --- | --- |
|  |  | **Baseline** | **1 month** | **3 months** | **6 months** |
| Cognitive Status | Six-item Screener | x |  |  |  |
| Sociodemographic and clinical characteristics | Ad hoc questionnaire | x |  |  |  |
| Comorbidities | CCI | x |  |  |  |
| Self-care maintenance, monitoring, management and self-efficacy | OSCI | x | x | x | x |
| Quality of life | Ostomy QoL | x | x | x | x |
| Depression | PHQ-9 | x | x | x | x |
| Adaptation, coping | OAI-23 | x | x | x | x |
| Stomal and peristomal complications | Ad hoc questionnaire | x | x | x | x |
| Number of improper accesses to health services | Ad hoc questionnaire | x | x | x | x |
| Lost working days | Ad hoc questionnaire | x | x | x | x |
| Mobility | Ad hoc questionnaire | x | x | x | x |

**Legend.** CCI, Charlson's Comorbidity Index; OAI-23, Ostomy Adjustment inventory-23; OSCI, Ostomy Self-care Index; QoL, Quality of Life.

**ATTACHMENT**

**Participating centres list**

| Coordinating Centre | Person in charge | E-mail |
| --- | --- | --- |
| Ambulatorio infermieristico per pazienti stomizzati - Policlinico Tor Vergata | Alessandro Sili | [alessandro.sili@ptvonline.it](mailto:alessandro.sili@ptvonline.it) |
| Satellite centres |  |  |
| Azienda SS. Antonio e Biagio e Cesare Arrigo - Alessandria | Bolgeo Tatiana | [tbolgeo@ospedale.al.it](mailto:tbolgeo@ospedale.al.it) |
| Istituto Tumori Pascale di Napoli | Maria Rosaria Esposito | [m.rosiespo@gmail.com](mailto:m.rosiespo@gmail.com) |
| Ambulatorio infermieristico per pazienti stomizzati – ASL Roma2 S. Eugenio – Pertini | Francesco Corvese | [fcorvese.uro@gmail.com](mailto:fcorvese.uro@gmail.com) |
| Ambulatorio infermieristico per pazienti stomizzati – ASL Roma6 | Cinzia Sandroni | [cinzia.sandroni@aslroma6.it](mailto:cinzia.sandroni@aslroma6.it) |
| Arcispedale s. Maria nuova - azienda ospedaliera di Reggio Emilia | Monica Guberti | [monica.guberti@ausl.re.it](mailto:monica.guberti@ausl.re.it) |
